# Hypercholesterolemia and Lymphatic Defects: The Chicken or the Egg?

**DOI:** 10.3389/fcvm.2021.701229

**Published:** 2021-06-23

**Authors:** Takuro Miyazaki, Akira Miyazaki

**Affiliations:** Department of Biochemistry, Showa University School of Medicine, Tokyo, Japan

**Keywords:** plasma dyslipidemia, LPA, S1P, calpain, nitric oxide synthase

## Abstract

Lymphatic vessels are necessary for maintaining tissue fluid balance, trafficking of immune cells, and transport of dietary lipids. Growing evidence suggest that lymphatic functions are limited under hypercholesterolemic conditions, which is closely related to atherosclerotic development involving the coronary and other large arteries. Indeed, ablation of lymphatic systems by Chy-mutation as well as depletion of lymphangiogenic factors, including vascular endothelial growth factor-C and -D, in mice perturbs lipoprotein composition to augment hypercholesterolemia. Several investigations have reported that periarterial microlymphatics were attracted by atheroma-derived lymphangiogenic factors, which facilitated lymphatic invasion into the intima of atherosclerotic lesions, thereby modifying immune cell trafficking. In contrast to the lipomodulatory and immunomodulatory roles of the lymphatic systems, the critical drivers of lymphangiogenesis and the details of lymphatic insults under hypercholesterolemic conditions have not been fully elucidated. Interestingly, cholesterol-lowering trials enable hypercholesterolemic prevention of lymphatic drainage in mice; however, a causal relationship between hypercholesterolemia and lymphatic defects remains elusive. In this review, the contribution of aberrant lymphangiogenesis and lymphatic cholesterol transport to hypercholesterolemic atherosclerosis was highlighted. The causal relationship between hypercholesterolemia and lymphatic insults as well as the current achievements in the field were discussed.

## Introduction

It is widely known that plasma dyslipidemia, which refers to elevation of plasma cholesterol and/or triglyceride levels, impacts chronic inflammatory diseases, such as type 2 diabetes mellitus and obesity. In particular, hypercholesterolemia can be responsible for lethal ischemic diseases, including acute coronary syndrome and stroke, which is the leading cause of death globally (1), as it is responsible for atherosclerotic vascular disease. Accordingly, controlling lipoprotein cholesterol is necessary for the primary and secondary prevention of ischemic diseases (2). The typical dyslipidemia pattern in type 2 diabetes comprises decreased high-density lipoprotein (HDL) cholesterol and, occasionally, elevated low-density lipoprotein (LDL) cholesterol levels (3). As a result, these metabolic diseases influence each other and worsen overall disease status. Oxidatively- and enzymatically-modified LDL can directly induce cytotoxic responses in blood vessels as well as metabolic organs. Much research has focused on such insults under the dyslipidemic conditions.

The lymphatic vessel network, which is spread throughout the body, is necessary for sustaining systemic fluid balance, intestinal absorption of fats, and draining waste products from the peripheral tissues. Lymphatic vessels, which are comprised of lymphatic endothelial cells (LECs), enable the production of chemokines to attract immune cells to the interstitial regions within the tissues (4), and interact with innate and adaptive immune cells. Among the LEC-derived chemokines, CCL21 has a pivotal role in the recruitment of dendritic cells (DCs) (5, 6). LECs can impact the adaptive immune system to regulate peripheral tolerance and immunomodulation (7). Microlymphatics around large blood vessels were discovered more than a 100 years ago (8). Notably, lymphatic dysfunction caused by Chy mutation or the deficiency of lymphangiogenesis factors reportedly potentiates atherosclerotic lesion progression (9, 10). This appears to be due to the limitation of reverse cholesterol transport. Recently, it was documented that hypercholesterolemia potentiates lymphangiogenesis around the atherosclerotic arteries, thereby modifying immune cell trafficking in lymphatic systems and atherosclerotic lesions (11). In contrast, the exact mechanisms underlying lymphatic insults as well as the causal relationship between hypercholesterolemia-driven lymphangiogenesis and atherosclerotic diseases are largely obscure. In this review, we focused on the relationship between hypercholesterolemia-driven lymphatic defects and atherosclerosis. The dysfunctional regulation of LECs under hypercholesterolemic conditions is also discussed.

## Lymphangiogenic Regulations Under the Hypercholesterolemic Conditions

In the late 18th century, Hoggan et al. discovered microlymphatics adjacent to the arterial wall (8). About a century later, it was reported that dysfunctional lymphatic drainage in allogenic transplanted hearts could be responsible for the progression of coronary atherosclerosis (12). More recently, it was reported that microlymphatics are enriched in the adventitial regions of human atherosclerotic plaques (13, 14). Notably, the density of the lymphatics is positively correlated with the severity of atherosclerosis; thus, it is thought that inflammatory elements, such as cytokines, chemokines, and growth factors, may induce lymphangiogenesis within atherosclerotic plaques (13). In addition to the adventitial regions, lymphatics appear to be detectable within the intraplaque regions of human carotid arteries (15). While some studies documented the enrichment of the lymphatics in atherosclerotic lesions, several other studies challenged this. Eliska et al. could detect microlymphatics in the periadventitial regions in human coronary arteries, whereas the vessels did not penetrate into the vascular walls even in normal and atheroprone arteries (16). Nakano et al. noted that microlymphatics in human coronary atheromas, which are mainly detectable in adventitial regions, were not correlated with the severity of atherosclerosis as well as the expression of lymphatic driver vascular endothelial growth factor-C (VEGF-C) and VEGF-D (17). *Apoe*-deficient hypercholesterolemic mice showed abundant adventitial lymphatics in comparison with age-matched wild-type mice, while these were reduced during the progression of the lesions (18). They interpreted that soluble VEGFR-2, which is upregulated in advanced atherosclerotic lesions, interrupts VEGF-C-induced lymphangiogenesis. Collectively, the drivers as well as mechanisms underlying the hypercholesterolemic regulation of periarterial microlymphatics, are currently unclear. Whereas the lymphatic regulation within other organs in hypercholesterolemic mice is mostly unclear, sinus lymphatic vessels within lymph nodes are shown to have hyperplastic appearance under hypercholesterolemic conditions (19).

## Lymphatic Systems and Atherogenesis

Atherosclerosis is a vascular disease in which cholesterol-enriched atherosclerotic lesions develop within the arterial intima, resulting in narrowing of the luminal vascular walls (20). Structural changes involving the arterial lumen lead to thrombus formation due to perturbation of laminar blood flow (21), resulting in arterial occlusion. In addition to blood clotting, arterial occlusion is induced via rupture of unstable atherosclerotic plaques (22). Vascular endothelial cell dysfunction is associated with the initiation of atherosclerosis (23). In the early stage of atherosclerosis, endothelial barrier functions are disrupted by environmental stressors, such as oxidative stress and inflammatory substances (24). This reduced barrier function is responsible for the infiltration of leukocytes and extravasation of plasma ingredients, including oxidative LDL. In particular, monocyte-derived macrophages tend to accumulate within the vascular walls. Monocytes normally patrol the circulatory system and are attracted by endothelial cell-derived chemoattractants, such as monocyte chemoattractant protein-1. After recruitment into the intimal space, monocytes differentiate into macrophages, which are further converted to cholesterol-enriched foamy macrophages (25). Since excessive accumulation of intracellular cholesterol induces cytotoxicity, deposition of LDL cholesterol in the vascular wall accelerates the necrosis of macrophages to form cholesterol-enriched unstable plaques, which contain large necrotic cores. During atherogenesis, lymphangiogenic drivers, including VEGF-C, appear to be enriched within atherosclerotic lesions (17, 18).

Recently, several studies have highlighted lymphatic defects under hypercholesterolemic conditions as well as the contribution of lymphatic systems to hypercholesterolemia (Table 1). Martel et al. documented the role of aortic microlymphatics in cholesterol drainage from atheromas (9). They transplanted atheroprone aortae derived from D6-cholesterol-loaded *Apoe*-deficient mice into *Apoe*-deficient recipient mice. The leakage of D6-cholesterol from the transplanted tissue was then monitored. As a result, lymphangiogenesis was detected within the connecting region between the recipient and implanted vessels, and D6-cholesterol was transferred to recipient mice. Both lymphangiogenesis and cholesterol drainage from the transplanted tissues were prevented via the administration of anti-VEGFR3 blocking antibody, suggesting that cholesterol drainage under hypercholesterolemic conditions is mediated through angiogenic microlymphatics. Vuorio et al. documented that insufficiency of lymphatic vessels by transgenic induction of sVEGFR3 or Chy mutant augments hypercholesterolemia in atherogenic mice (10). They crossed low-density lipoprotein receptor/apolipoprotein B48-double knockout mice with sVEGFR3- transgenic mice or Chy-mutant mice to induce lymphatic insufficiency in hypercholesterolemic mice. As a result, sVEGFR3-induced lymphatic insufficiency facilitated the progression of atherosclerotic lesions. Rademakers et al. reported that dissection of microlymphatics, which connect the carotid artery and regional lymph nodes, resulted in the expansion of carotid atherosclerotic lesions (11). Since surgical lymphatic dissection appears to induce accumulation of CD3^+^ T cells, but not macrophages, adventitial microlymphatics may be a route for emigrating CD3^+^ T cells from the lesions. Collectively, lymphatic insufficiency exacerbates atherosclerotic lesion progression within arteries.

**Table 1:** Lymphatic function-related assessment and atherosclerosis-related phenotypes in normocholesterolemic and hypercholesterolemic animal models.

**Animal or surgical procedure**	**Lesion size**	**Plasma cholesterol levels**	**Other phenotype**	**References**
Wildtype mice with surgical dissection of tail lymphatics	N/A	N/A	Cholesterol transport from tail to plasma↓	(9)
Chy mice	N/A	↑	Reverse cholesterol transport↓	(9)
Transplantation of D6-cholesterol-loaded atheromas to Apoe^−/−^ mice	N/A	N/A	Cholesterol transport from transplanted atheromas to plasma↓	(9)
Chy/Ldlr^−/−^/ApoB^100/100^ mice with Western diet	→	↑	Intraplaque lymphatics↓	(10)
sVEGFR3 × Ldlr^−/−^/ApoB^100/100^ mice with Western diet	↑	↑	Intraplaque lymphatics↓	(10)
Apoe^−/−^ mice with VEGF-C treatment	N/A	→	Reverse cholesterol transport↑	(26)
Wildtype mice with surgical disruption of afferent lymphatic vessels	N/A	→	Reverse cholesterol transport↓	(26)
Pcsk9^−/−^/Ldlr^−/−^ mice	N/A	↓	Lymphatic drainage (collecting vessels)↑ Intimal microlymphatics in atheromas → Plasma and lymph HDL cholesterol↓	(27)
Ldlr^−/−^/hApoB^100/100^ mice with VEGF-C^152S^ treatment	N/A	↑	Lymphatic drainage (collecting vessels)↑ Intimal microlymphatics in atheromas→	(27)
Apoe^−/−^ mice (Chow vs Western diet)	N/A	N/A	Intraplaque lymphatics↓ VEGF-C in aortic wall↑ sVEGFR-2 in aortic wall↑	(18)
Ldlr^−/−^ mice with apoA-I treatment and Western diet	↓	↓	Lymphatic transport↑ Periaortic and dermis microlymphatics↑ Lymphatic fluid leakage↓	(28)
Apoe^−/−^ mice with surgical dissection of plaque-associated lymphatic vessels	↑	N/A	Intraplaque CD3+ T cells↑	(11)
Apoe^−/−^ mice with transfection of soluble hVEGFR3	→	N/A	Intraplaque CD3+ T cells↑	(11)
LDLR^−/−^ regression model with VEGF-C^152S^ treatment	↓	↓	Lymphatic transport↑ Periaoritic microlymphatics → Intraplaque CD68+ macrophages↓ Contraction of collecting lymphatic vessels↑ FOXC2 in collecting lymphatic vessels↑	(29)

*→Unchanged, ↑Increased, ↓Decreased*.

## Lymphatic Vessels and Reverse Cholesterol Transport

Reverse cholesterol transport is indispensable for the clearance of excessive cholesterol from peripheral tissues (30). Mechanistically, ATP-binding cassette transporter A1 and ATP-binding cassette transporter G1 in peripheral tissues integrate intracellular cholesterol into apolipoprotein A-I (ApoA-I), a constitutive apolipoprotein of HDL, thereby generating HDL cholesterol. Subsequently, plasma and lymph HDL cholesterol is incorporated into hepatocytes through scavenger receptor B1 (SR-B1), and HDL receptors (31). Accordingly, defective reverse cholesterol transport induces elevation of plasma cholesterol levels, leading to severe hypercholesterolemia. Some studies have documented that lymphatic dysfunction leads to defective reverse cholesterol transport. Hypercholesterolemia in *Apoe*-deficient mice reportedly abrogates lymphatic drainage of intravenously injected HDL as well as its transport to the liver (26). SR-B1 in LECs plays a central role in lymphatic reverse cholesterol transport, since this molecule can mediate transcytosis of HDL in the cells (26). Furthermore, administration of ApoA-I in hypercholesterolemic mice suppressed atherosclerosis development, accompanied by resolution of lymphatic hyperpermeability involving collecting lymphatic vessels (28). While ApoA-I upregulates VEGFR3 in cultured LECs, the exact mechanisms by which ApoA-I retains lymphatic function remain elusive. Since the VEGF-C/VEGFR3 signaling pathway reportedly increase permeability in LECs (32), causal relationship between VEGFR3 and the ApoA-I-induced lymphatic protection is unknown. In addition to VEGFR3 induction, ApoA-I appears to potentiate platelet adhesion to LECs. Platelets are necessary for blood/lymphatic vessel separation during embryogenesis (33) and maintenance of the lymphvenous junction throughout life (34). Since platelets reportedly confer stability to LECs through physical interaction between C-type lectin-like receptor 2 in platelets and podoplanin in LECs, it is speculated that ApoA-I-induces platelet adhesion to LECs, stabilizing lymphatic vessels. Similar to ApoA-I, deficiency of proprotein convertase subtilisin/kexin type 9 (PCSK9), a negative regulator of LDL receptor, recovered lymphatic drainage in hypercholesterolemic mice concomitantly with the suppression of atherosclerotic lesions (27). Indeed, the LDL receptor, which is expressed in LECs in mice, is downregulated in hypercholesterolemic mice concomitantly with elevation of plasma PCSK9 levels. Consistently, downregulated LDL receptors in LECs are recovered via the targeted deficiency of PCSK9 (27). Retaining LDL receptors in LECs was not associated with the density of lymphatic vessels. Thus, LDL receptors may contribute to lymphatic integrity rather than lymphangiogenic functions. Furthermore, administration of ezetimibe, a cholesterol-lowering drug, in hypercholesterolemic mice retained lymphatic drainage (26). These observations suggest that hypercholesterolemia, accompanying with declining plasma HDL-associated ApoA-I, may disrupt the integrity of LECs and their barrier functions, particularly in large lymphatic vessels, concomitantly with dysfunction of SR-B1 in microlymphatics.

Since lymphatic insufficiency induced by transgenic overexpression of sVEGFR3 reportedly elevated plasma cholesterol levels in hypercholesterolemic mice, particularly regarding VLDL and LDL fractions (10), it is likely that endogenous VEGF-C and/or VEGF-D signaling pathways may contribute to the clearance of these lipoproteins even under hypercholesterolemic conditions. Lymphatic vessels are reportedly associated with endogenous lipoprotein metabolism through VLDL and LDL, as well as intestinal absorption of chylomicrons (35). Therefore, VEGFR3-mediated lymphatic modulation may be attributed to cholesterol transport toward peripheral tissues through VLDL and LDL. Since VLDL and LDL modification by lymphatic insufficiency appear to be independently of HDL (10), these actions are probably independent of HDL-associated reverse cholesterol transport. In addition to overexpressing soluble VEGFR3, targeted deficiency of VEGF-D exacerbates hypercholesterolemia (36). Indeed, VEGF-D deficiency substantially elevates plasma cholesterol, particularly in chylomicron and chylomicron remnant fractions. In this case, VEGF-D appears to contribute to the hepatic transcriptional regulation of lipid handling elements as well as the incorporation of chylomicron remnants into the liver, independent of its lymphangiogenic effects. Accordingly, VEGF-D deficiency did not accelerate atherosclerosis progression in hypercholesterolemic mice. Hence, lymphangiogenic factors, at least VEGF-D, may have pleiotropic lipomodulatory functions, in addition to their lymph-modulatory functions. Collectively, hypercholesterolemia appears to abrogate transcytosis of HDL within microlymphatics as well as integrity in large lymphatic vessels as note above, whereas the contribution of periarterial microlymphatics to atherogenesis and the exact pathophysiologic mechanisms underlying the hypercholesterolemic lymphatic insults are largely unknown.

## Lymphangiogenesis Under Hypercholesterolemia

VEGF-C is a robust lymphangiogenic factor, which is indispensable for lymphatic development (37). Several lines of evidence suggest that VEGF-C is associated with lymphatic patterning under hypercholesterolemic conditions. In addition to VEGF-C, several modulatory elements that can be involved in lymphangiogenesis under hypercholesterolemia have been reported to date.

### VEGF-C/VEGF-D Signaling

VEGF-C is abundantly expressed in foamy macrophages and smooth muscle cells in human coronary atherosclerotic lesions (17). Furthermore, VEGF-C is reportedly elevated in moderate to advanced atheromas involving hypercholesterolemic mice (18). However, it is noteworthy that adventitial microlymphatics regress during lesion progression. Targeted delivery of VEGF-C to atherosclerotic lesions failed to induce adventitial lymphangiogenesis (38). Accordingly, the association between VEGF-C accumulation during atherogenesis and periarterial lymphangiogenesis remains unclear. Pretreatment of hypercholesterolemic mice with VEGF-C reportedly improved the contraction rate of collecting lymphatic vessels and lymphatic transfer of inflammatory cells, thereby suppressing atherosclerotic lesions (29). This suggests that VEGF-C may impact the trafficking of immune cells via the collecting vessels under hypercholesterolemic conditions rather than through prolymphangiogenic actions in periarterial regions.

VEGF-D has a close structural and functional similarity to VEGF-C (39) and possesses robust angiogenic and lymphangiogenic activity. Similar to VEGF-C, VEGF-D action is mediated through VEGFR3 (40), though it does not play a major role in lymphatic development in mice (41). As noted above, targeted deficiency of VEGF-D in hypercholesterolemic mice elevated plasma cholesterol and triglyceride levels without altering lymphangiogenesis and atherosclerosis (36). Transgenic induction of soluble VEGFR3, which can interrupt VEGF-C/D-induced signaling, was unable to inhibit adventitial lymphangiogenesis (11). Hence, the lymphangiogenic effects of VEGFR3 signaling under hypercholesterolemic conditions remain elusive.

### Sphingosin-1-Phosphate Signaling

Sphingosine-1-phosphate (S1P) is a bioactive lipid synthesized from ceramide (42). In the initial step of its synthesis, ceramidase converts ceramide into sphingosine. Subsequently, sphingosine kinase (Sphk)1 and Sphk2 phosphorylate sphingosine to form S1P (43). Five subtypes of S1P receptors, including S1PR1- S1PR5, have been reported to be involved in various physiological and pathophysiological events, such as cardiovascular regulation, immune regulation, neurodevelopment, neuroprotection, and fibrogenic responses (44). Among them, S1PR1 was originally cloned from vascular endothelial cells (45). Notably, S1P is abundantly carried on HDL (46) and is believed to contribute to the antiatherogenic action of HDL (47). *Lyve1*-driven ablation of Sphk1 and lacking Sphk2 interrupt the production of S1P in LECs, thereby depleting S1P within the lymph, but not in the plasma (48). Such lymphatic S1P depletion induces aberrant lymphatic morphology involving the trachea and diaphragm, and interrupts lymphocyte egress from peripheral tissues, indicating that LEC-derived S1P plays a crucial role in lymphatic patterning and lymphocyte trafficking. Consistently, S1P is reportedly involved in the transmigration of lymphocytes in LECs through S1PR2/ERK-mediated regulation of junctional proteins, including VE-cadherin, occludin, zonulin-1, and VCAM1 expression (49). Importantly, S1P has been shown to decline in LNs of hypercholesterolemic mice (19). Moreover, hypercholesterolemia appears to accelerate lymphangiogenesis within LNs and impair lymphocyte egress from these LNs. Although intervention of S1P signaling in hypercholesterolemic mice has not been performed so far, S1P depletion in hypercholesterolemic mice can impact lymphatic pattering and lymphocyte trafficking.

### Lysophosphatidic Acid Signaling

Lysophosphatidic acid (LPA), a multifunctional bioactive lysophospholipid, is involved in the pathogenesis of atherosclerosis (50–53). LPA can be generated via several enzymatic pathways (54). For instance, LPA is synthesized from lysophosphatidylcholine by the action of autotaxin (lysophospholipase D). Alternatively, phospholipase A2 mediates the conversion of phosphatidic acid to LPA through hydrolysis of its sn-2 acyl chain. In addition to the synthetic pathways noted above, LPA can be derived from glycerol-3-phosphate by glycerol-3-phosphate acyltransferase-induced addition of fatty acids toward the sn-1 position. Currently, six species of G-protein-coupled LPA receptors, LPA1-LPA6, have been reported (51). Among these receptors, LPA4 is involved in lymphatic vessel formation during embryogenesis (55). Moreover, LPA contributes to NF-κB-mediated induction of IL-8 in human dermal LECs, thereby potentiating lymphangiogenesis (56). Furthermore, it has been documented that LPA1 is coupled with S1PR1 to induce S1PR1/β-arrestin coupling and inhibit Gαi signaling (57). This potentiates the disorganization of intercellular junctions in sinus-lining LECs within LNs, which facilitates the lymphatic transfer of lymphocytes toward the LNs. Of note, LPA reportedly accumulates within atherosclerotic lesions in human and hypercholesterolemic mice (58, 59). While the prolymphangiogenic roles of LPA in hypercholesterolemic conditions have not been proved in human and animal experimental models, it is speculated that LPA in atheromas attracts adjacent LECs to induce lymphangiogenesis.

### Nitric Oxide Signaling

Nitric oxide (NO) is primarily derived from vascular endothelial cells through endothelial NO synthase (eNOS) and its substrate L-arginine (60), and is associated with vasodilation when adjusting regional pressure-flow balance (61–63). In addition to its vasomotor functions, NO possesses robust angiogenic effects on vascular endothelial cells (64, 65). Mechanistically, treatment of vascular endothelial cells with NO leads to cGMP-dependent activation of protein kinase G through guanylate cyclase to upregulate target molecules (66). Furthermore, NO stabilizes hypoxia-inducible factor-1α and the subsequent production of VEGF-A (67). Importantly, VEGF-C activates eNOS to generate NO in LECs. Indeed, treatment of human dermal LECs with VEGF-C potentiates lymphangiogenesis through the activation of eNOS (68). Targeted deficiency of eNOS or pharmacological inhibition of eNOS suppresses peritumoral lymphatic hyperplasia (68). Moreover, treatment of LECs with NO donors potentiates lymphangiogenic tube formation concomitantly with elevation of cGMP levels in affected cells (69). Singla et al. identified matrix protein R-spondin 2 (RSPO2) as a lymphangiogenesis inhibitor (70). RSPO2 expression was noted in vascular endothelial cells and LECs, which counteracted the VEGF-C-induced activation of AKT, resulting in the depletion of NO and subsequent lymphangiogenesis. Since a variety of vasoactive elements can potentiate NO production, it is possible that perturbation of these elements under hypercholesterolemic conditions disturbs lymphangiogenesis.

There is another possibility of NO-mediated pathogenic lymphatic modifications. Liao et al. documented that NO derived from iNOS-overexpressing CD11b+ myeloidal DCs, which accumulated around the subcutaneous lymphatic vessels during oxazolone-induced contact sensitization, could weaken rhythmic lymphatic contraction (71). Intriguingly, lymphatic contraction appears to be associated with T cell activation as well as the pathogenesis of oligodendrocyte glycoprotein peptide 35–55-induced autoimmune encephalomyelitis (71), suggesting that lymphatic contraction has an immunomodulatory role during inflammation. While VEGF-C reportedly improves contractile functions in collecting lymphatic vessels, as noted above (29), there is no evidence of perilymphatic accumulation of DCs and aberrant contractile regulation of collecting lymphatic vessels under hypercholesterolemic conditions. Hence, future studies should investigate NO homeostasis and immune cell composition around large lymphatic vessels.

### Calpain Systems

Calpain, a superfamily of intracellular Ca^2+^-dependent proteases, can be defined as molecules possessing a calpain-like cysteine protease sequence (CysPc) motif. Currently, 15 species of calpain isozymes have been identified in mammals (72, 73). Among the calpain subtypes, calpain-1 and calpain-2 are classified into conventional calpains and comprise a heterodimer of the small regulatory subunit CAPNS1 and their unique catalytic subunits CAPN1 and CAPN2, respectively (74). Conventional calpains can be intracellularly activated in response to various physiological and pathogenic stressors, such as lysophospholipids, hypoxia, cytokines, and growth factors, thereby proteolyzing functional cellular proteins through limited proteolytic cleavage (74, 75). As a result of proteolysis, calpain enables the modification of cellular functions and phenotypes. Our previous study showed that conventional calpains in vascular endothelial cells are involved in pathological angiogenesis in oxygen-induced retinopathy and cancer allograft models in mice (76). Mechanistically, activation of the conventional calpains in vascular endothelial cells elicits shedding of suppressor of cytokine signaling protein 3, resulting in the sensitization of Janus kinase/signal transducer and activators of transcription systems. Importantly, these inflammatory signaling pathways are associated with VEGF-C production. Since VEGF-C accumulatively accelerates VEGF-A-induced angiogenic responses in vascular endothelial cells (77), the calpain-activated cytokine signaling can synergize with VEGF-A-driven signaling cascades. As a result, calpain overactivation accelerates the aforementioned proliferative insults in mice. Of note, calpain-2 potentiates lymphangiogenesis in human dermal LECs through NO production (78). Importantly, conventional calpains can be upregulated by lysophosphatidylcholine under hypercholesterolemic conditions (79). Thus, it is speculated that these molecules are activated even in LECs under hypercholesterolemic conditions. In contrast to the conventional calpains, the contribution of unconventional calpains to lymphangiogenesis has not been reported so far, although they are reportedly involved in a variety of physiological and pathophysiological events (80–82).

## Causal Relationship between Lymphatic Insults and Hypercholesterolemia

**Figure 1:**
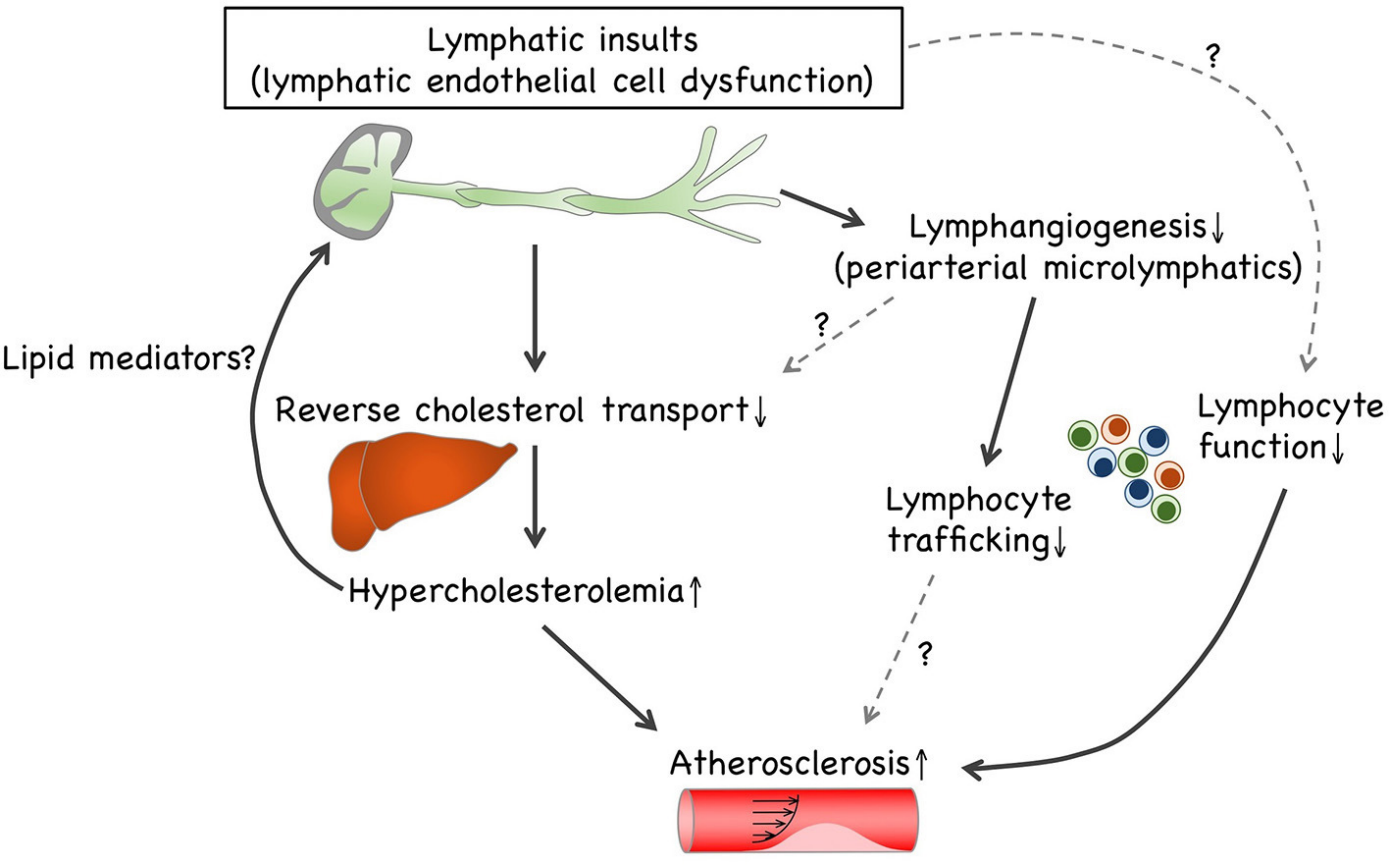
Causal relationship between lymphatic insults and hypercholesterolemia. Lymphatic insults under hypercholesterolemic conditions are likely to interrupt reverse cholesterol transport, which can be owed to the turbulence of lymphatic drainage within collecting lymphatic vessels and to impaired lymphangiogenesis involving periaortic microlymphatics. Additionally, lymphangiogenesis is disrupted under hypercholesterolemic conditions independently of VEGF-C and VEGF-D depletion. Such lymphangiogenic insufficiency can be responsible for limited lymphocyte trafficking. LECs enable exerting antigen presentation to modulate dendritic cells and T cells, thereby modifying adaptive immunity. Therefore, hypercholesterolemic lymphatic insults presumably cause lymphocyte dysfunction that accelerates atherosclerosis progression. In addition, several dyslipidemic lipid mediators, such as lysophospholipids, enable modification of lymphatic patterning and functions. Since cholesterol-lowering trials reportedly recover hypercholesterolemic lymphatic insults, it is possible that hypercholesterolemia can precede lymphatic insults, presumably owing to lipid mediators.

Growing evidence suggest that lymphatic defects under hypercholesterolemic conditions are likely to interrupt reverse cholesterol transport (Figure 1). This may be due to dysfunctional HDL transcytosis in microlymphatics and impaired lymphatic drainage involving collecting lymphatic vessels. To the best of our knowledge, impaired cholesterol transport is likely to be the primary cause of atherosclerosis modification by lymphatic insults. Concomitantly with the prevention of reverse cholesterol transport, lymphangiogenesis is disrupted under hypercholesterolemic conditions. Such lymphangiogenic insufficiency is unlikely to be dependent on the depletion of VEGF-C and VEGF-D. Although impaired lymphangiogenesis can be responsible for the limitation of lymphocyte trafficking, further research is needed to investigate the relationship between limited lymphocyte trafficking and immune responses, such as regulatory T cell-driven immunosuppression. It is noteworthy that LECs reportedly exert antigen presentation to modulate DCs and T cells, thereby modifying adaptive immunity (7), which presumably causes lymphocyte dysfunction to accelerate atherosclerosis progression. In addition, several dyslipidemic lipid mediators, such as lysophospholipids, modify the lymphatic structure and function. Several lipid-lowering drugs and apoA-I are reportedly effective in improving lymphatic functions, including lymphatic drainage and reverse cholesterol transport; accordingly, it is possible that hypercholesterolemia can precede lymphatic insults, which is presumably mediated through dyslipidemic lipid mediators. Hence, future investigations are necessary to explore the causal relationship between pathophysiologic lymphatic regulation and hypercholesterolemia-driven atherogenesis.

## Future Directions

A growing body of evidence suggests that lymphatic defects are responsible for hypercholesterolemia. At the same time, hypercholesterolemia itself appears to elicit lymphatic insults. In addition, several investigations support the notion that lymphangiogenesis around the atheroprone artery exerts lymphocyte migration from atherosclerotic lesions, which can be sustained by VEGF-C. Since the lymphangiogenic role of VEGF-C in periarterial regions has been challenged by some researchers, exploring alternative lymphatic drivers under hypercholesterolemic conditions is necessary. While several bioactive lipids enable the induction of lymphangiogenesis, lipid composition in the lymphatic environment under hypercholesterolemic conditions has not been fully elucidated. Thus, defining lipid composition and its temporal changes in the lymphatic environment during hypercholesterolemia is a promising approach for understanding the causal relationship. In addition to the research exploring environmental factors that induce hypercholesterolemic lymphatic insults, pathogenic intracellular mechanisms in LECs should be investigated in future studies. Employing Cre-loxP systems enables the elucidation of the effects of lymphatic defects on atherogenesis in mice. Defining lipid compositions and intracellular mechanistic insight will be helpful for developing a strategy for the prevention of atherosclerotic diseases and for estimating the personal susceptibility to atherosclerotic diseases.

## Author Contributions

TM wrote the manuscript. AM revised the manuscript. Both authors contributed to the article and approved the submitted version.

## Conflict of Interest

The authors declare that the research was conducted in the absence of any commercial or financial relationships that could be construed as a potential conflict of interest.
